# Medical cannabis use in Australia seven years after legalisation: findings from the online Cannabis as Medicine Survey 2022–2023 (CAMS-22)

**DOI:** 10.1186/s12954-024-00992-1

**Published:** 2024-05-28

**Authors:** Llewellyn Mills, Jonathon C. Arnold, Anastasia Suraev, Sarah V. Abelev, Cilla Zhou, Thomas R. Arkell, Iain S. McGregor, Nicholas Lintzeris

**Affiliations:** 1 Specialty of Addiction Medicine, Faculty Medicine and Health, University of Sydney, 591 South Dowling Street, Surry Hills, NSW 2010 Australia; 2 Drug and Alcohol Services, South East Sydney Local Health District, Sydney, NSW Australia; 3 Drug and Alcohol Clinical Research and Improvement Network (DACRIN), Sydney, NSW Australia; 4 Lambert Initiative for Cannabinoid Therapeutics, The University of Sydney, Sydney, NSW Australia; 5 Faculty of Science, School of Psychology, The University of Sydney, Sydney, NSW Australia; 6 Brain and Mind Centre, The University of Sydney, Sydney, NSW Australia; 7 Centre for Human Psychopharmacology, Swinburne University of Technology, Hawthorn, VIC Australia

**Keywords:** Cannabis, Medical cannabis, Medicinal cannabis, Consumer survey

## Abstract

**Background:**

Cannabis was legalised for medical purposes in 2016. Uptake was initially slow, but since 2019 there has been a large increase in the number of Australians who have been prescribed cannabis for medical reasons. Yet a significant number of consumers continue to treat their medical conditions via illicitly-sourced cannabis. Little is known about how these two groups of medical cannabis consumers differ.

**Methods:**

The anonymous Cannabis-As-Medicine Survey 2022–2023 (CAMS-22) was available for completion online from December 2022 to April 2023 to adult Australians who had used cannabis to treat a medical condition in the previous year. Recruitment occurred through social media, consumer forums, and medical practices. Questions included demographic characteristics, patterns of cannabis use, conditions treated, and self-rated effectiveness.

**Results:**

Of the 3323 respondents included in these analyses, 2352 (73%) mainly used prescribed medical cannabis, 871 (27%) mainly used illicit. Prescribed users were significantly more likely than illicit users to have had their health condition diagnosed (OR = 1.7, 95% CI 1.3, 2.2), to consume their cannabis via oral (OR = 1.9; CI 1.5, 2.4) or vaporised (OR = 5.2; CI 4.0, 6.8) routes, and to be sure of the composition of their medical cannabis (OR = 25.0; CI 16.7, 50.0). Prescribed users were significantly less likely to have used cannabis non-medically before medical use (OR = 0.6, CI 0.5, 0.7), consume cannabis via smoked routes (OR = 0.2, CI 0.1, 0.2), and to report any side effects (OR = 0.1; CI 0.1, 0.2). The most common conditions among both prescribed and illicit users were pain (37%), mental health (36%), and sleep (15%) conditions. Prescribed users were significantly more likely to use cannabis to mainly treat a pain (OR = 1.3; CI 1.1, 1.5) or sleep condition (OR = 1.4; CI 1.1, 1.7) and less likely to treat a mental health condition (OR = 0.8; CI 0.7, 0.9). There were no between-group differences in effectiveness with 97% saying medical cannabis had improved their symptoms.

**Conclusions:**

From a harm-reduction perspective there is much to recommend prescribed medical cannabis; it has fewer side-effects than illicit, is used more safely (oral or vaporised versus smoked routes), gives consumers greater certainty regarding the composition and quality of their medicine, and does not risk exposure to the criminal justice system. Of concern, however, is the apparent willingness of prescribers to prescribe for indications for which there is limited evidence of efficacy, such as mental health and sleep conditions.

**Supplementary Information:**

The online version contains supplementary material available at 10.1186/s12954-024-00992-1.

## Introduction

Research into the efficacy of medical cannabis for a range of health conditions has grown rapidly in recent years [1, 2]. As a result governments around the world have begun to relax the laws restricting medical use of cannabis. Sixty-four countries now allow some form of legal medical cannabis use, with considerable variety across jurisdictions in the products available, how they are accessed, and the way they can be prescribed by doctors [3]. Legal access to medical cannabis was introduced in Australia in 2016, and since then the Australian medical cannabis landscape has changed dramatically. Before 2016, Australians who wished to use cannabis as a medicine had to source it illegally, there was little quality control in the composition of cannabis products, and consumers used their cannabis-based medicines without medical guidance [4, 5]. As of 2024, patients have had access to more than 800 different medical cannabis products [6] to treat any type of medical condition [7–10] via the Special Access (SAS) and Authorised Prescriber (A-P) Schemes of the Therapeutic Goods Administration (TGA), the Australian government’s regulatory body for medicines [9].

A number of changes that support clinical practice have occurred in recent years, including publication of clinical guidance, educational material, and professional development programs [11–14]. Processes allowing doctors to prescribe medicinal cannabis have also been streamlined by the TGA and there is no credentialling process or mandatory training requirements for medical practitioners, who may prescribe cannabis for any clinical indication they deem appropriate under the SAS-B scheme. As a result, there has been steady growth in the number of patients being prescribed medical cannabis, as gauged by the number of SAS-B approvals issued by the TGA. Between 100,000 and 120,000 SAS-B approvals were granted each year in 2021–2023. In parallel, there has been extraordinary recent growth in prescriptions under the A-P scheme, with medical cannabis products dispensed under the A-P now approximately fivefold greater than those under SAS-B [15]. Overall, it is thought that that hundreds of thousands of Australians have accessed prescribed medical cannabis since 2016 [16] with estimates of a tenfold increase since 2019 in the number of users who are receiving their cannabis exclusively via doctor prescription [17]. Like medical cannabis consumers in other jurisdictions around the world [18–20], SAS-B data indicates that the conditions Australians most commonly treat with medical cannabis are chronic pain, anxiety, and sleep disorders [16].

The dramatic increase in prescriptions reflects changing attitudes among doctors towards medical cannabis. Recent surveys and meta-analyses indicate that health professionals in Australia and the rest of the world are increasingly positive in their attitudes towards medical cannabis [21–25]. However, many clinicians remain concerned about their lack of experience prescribing; the increase in prescribing for conditions where there is limited empirical evidence for efficacy, such as mental health and sleep conditions; and the potential for harm to patients and society generally due to inappropriate prescribing [24–28].

Regulatory changes, emerging evidence, increases in the number of health practitioners providing medical cannabis treatment, and expansion in the range of cannabis products available since 2016 have resulted in a changing medical cannabis landscape for consumers. Consumer experiences over this time have been tracked using the biennial Cannabis as Medicine Survey (CAMS) undertaken by our group. These anonymous surveys commenced in 2016, just prior to legalisation (CAMS-16 [4]), and were followed by CAMS-18 [29], CAMS-20 [30] and, in the present report, CAMS-22. Strikingly, very few respondents in the CAMS-16 or CAMS-18 surveys had been prescribed cannabis products (< 1% and 2.4% respectively) and these early surveys showcased a largely hidden population of Australians who were self-medicating with illicit cannabis supplies. The CAMS-20 survey (in 2020) showed a substantial number of respondents (38% [601/1600]) were using prescribed medical cannabis, enabling a comparison of the differing characteristics and experiences of prescribed versus illicit users. This revealed that prescribed users were significantly (i) more likely to treat a pain condition and less likely to treat a mental health condition; (ii) more likely to consume their medical cannabis via oral than smoked routes, and (iv) less likely to have used cannabis before being prescribed it medically [30].

The CAMS-22 survey was launched in December 2022, following several years of rapid growth in numbers of TGA approvals, suggesting a relatively mature medical cannabis landscape compared to prior surveys. Accordingly, the overall aim of this paper was to present the top-level results of the CAMS-22 survey, with a focus on the experiences and patterns of use of medical cannabis users and on differences between illicit versus prescribed medical cannabis use.

## Methods

Similar to previous CAMS surveys [4, 29, 30], CAMS-22 was a cross-sectional, anonymous, online survey of Australian adults self-reporting cannabis use for medical purposes in the previous year. The survey asked respondents to report, among other things: their demographic characteristics; the medical conditions they treated with medical cannabis; their current and lifetime patterns of medical and non-medical cannabis use; where they obtain and how they consume their medical cannabis; the cannabinoid profile of the medical cannabis they consume [e.g. proportion of tetrahydrocannabinol (THC) vs. cannabidiol (CBD)]; the perceived effectiveness of their medical cannabis; and their experiences accessing medical cannabis in the current regulatory environment (full survey questionnaire available in Additional file 1).

Data were collected and stored via Research Electronic Data Capture (REDCap), a web-based software platform for administering surveys [31], and the study was promoted through social media, consumer group web pages, at conferences, and through a number of private medical cannabis clinics. Eligibility criteria were: (a) informed consent, (b) aged ≥ 18 years, (c) self-identified as using cannabis or a cannabis-based product for a medical purpose within the previous 12 months, and (d) resident in Australia. The survey was available online from the 16th of December 2022 to the 20th of April 2023.

Our focus in the primary analysis was to examine differences between illicit medical cannabis users and prescribed medical cannabis users. ‘Prescribed’ users were those respondents who reported prescribed cannabis as their main (or only) source of medical cannabis in the preceding 12 months, whereas ‘illicit’ users reported mainly sourcing their medical cannabis illicitly.

We used single-level regressions to analyse the data, with main source of medical cannabis (illicit vs prescribed) as the sole predictor: Gaussian regression for continuous outcomes (e.g. age, cost), binary logistic regression for two-level categorical outcomes (e.g. relationship status, education), multinomial logistic regression for greater than two-level outcomes (e.g. main condition treated, side effects), aggregated binomial regression for bounded count data (e.g. number of side-effects reported) and cumulative link models for ordinal outcomes (e.g. change in tobacco, alcohol, or medication use). The Benjamini–Hochberg procedure was used to control the family-wise error rate [32]. All analyses were available-case with no imputation of missing values. Statistical analyses were performed in R version 4.2.2 [33] using the tidyverse [34], nnet [35], ordinal [36] and emmeans [37] packages.

## Results

### Participants

A total of 5892 respondents commenced the survey. Of these, 325 did not provide consent, 522 did not proceed beyond the consent page, 5 were deemed as not providing serious responses to questions, and 2 were test responses completed by researchers, leading to 854 exclusions in total. Our focus in this analysis was on differences between prescribed and illicit users. For structural reasons we placed the questions concerning prescribed vs illicit use in the latter half of the survey. Because of this, the 1815 respondents who did not progress far enough through the survey to answer these questions (out of 5038 who commenced the survey) were excluded. This left 3223 respondents to include in the full analysis. In total, 871 of these 3223 (27%) respondents indicated that in the last year their only or main source of medical cannabis was illicit and 2352 (73%) indicated prescribed. A total of 374 of the 3223 respondents indicated they were currently using both prescribed and illicit medical cannabis. Of these, 271/374 (73%) indicated they mainly used prescribed and 103/374 (28%) mainly illicit.

### Demographics

Respondents’ demographic characteristics and tobacco and alcohol use are reported in Table 1. Prescribed medical cannabis users were significantly more likely to be male (OR = 1.6, CI 1.3, 2.0), in a relationship (OR = 1.4, CI 1.2, 1.6), have attained a tertiary qualification (OR = 1.4, CI 1.2, 1.7), employed (OR = 1.6, CI 1.4, 1.9), and not use tobacco (OR = 2.4, CI 1.9, 3.0) than illicit users. Respondents mostly heard about the survey through social media (42%, 1353/3223), though a notable proportion were recruited through online forums (16%, 508/3223) and medical cannabis providers (14%, 451/3223).

**Table 1:** Demographic characteristics and other substance use among medical cannabis users

Characteristic	Prescribed	Illicit	Total	Comparisons estimate (95% CI)^a^
Age, median (IQR), numeric (range:18–87)	42 (33,52)	42 (30,55)	42 (33,53)	P–I: − 0.2 (− 1.2, 0.8)
Gender, categorical polytomous, n (%)				
Female	794/2352 (34%)	386/871 (44%)	1180/3223 (37%)	**P/I: 0.6 (0.5, 0.8)**
Male	1469/2352 (63%)	447/871 (51%)	1916/3223 (59%)	**P/I: 1.6 (1.3, 2.0)**
Other^b^	89/2352 (4%)	38/871 (4%)	127/3223 (4%)	P/I: 0.9 (0.5, 1.5)
In relationship^c^, categorical binary, n (%)	1519/2352 (65%)	494/871 (57%)	2013/3223 (63%)	**P/I: 1.4 (1.2, 1.6)**
Aboriginal and/or Torres Strait Islander, categorical binary, n (%)	99/2352 (4%)	64/871 (7%)	163/3223 (5%)	**P/I: 0.6 (0.4, 0.8)**
Attained tertiary qualification^d^, categorical binary, n (%)	1859/2352 (79%)	631/871 (72%)	2490/3223 (77%)	**P/I: 1.4 (1.2, 1.7)**
Employed^e^, categorical binary, n (%)	1423/2352 (61%)	426/871 (49%)	1849/3223 (57%)	**P/I: 1.6 (1.4, 1.9)**
How respondents heard of survey^f^, categorical polytomous, n (%)				
Social media	799/2352 (34%)	554/871 (64%)	1353/3223 (42%)	**P/I: 0.3 (0.2, 0.4)**
Online forum	391/2352 (17%)	117/871 (13%)	508/3223 (16%)	**P/I: 1.3 (1.0, 1.7)**
Medical cannabis provider	415/2352 (18%)	36/871 (4%)	451/3223 (14%)	**P/I: 5.0 (3.3, 7.4)**
Private cannabis clinic	44/2352 (1%)	10/871 (1%)	54/3223 (2%)	P/I: 1.6 (0.8, 3.6)
Doctor/healthcare provider	21/2352 (1%)	9/871 (1%)	30/3223 (1%)	P/I: 0.9 (0.4, 2.1)
Other sources	682/2352 (29%)	145/871 (17%)	827/3223 (26%)	**P/I: 2.0 (1.6, 2.6)**
Tobacco use in previous 28 days, categorical polytomous, n (%)				
None	1821/2351 (78%)	511/871 (59%)	2332/3222 (72%)	**P/I: 2.4 (1.9, 3.0)**
Less than daily	162/2351 (7%)	91/871 (10%)	253/3222 (8%)	**P/I: 0.6 (0.4, 0.9)**
Daily	368/2351 (16%)	269/871 (31%)	637/3222 (20%)	**P/I: 0.4 (0.3, 0.5)**
Alcohol use in previous 28 days, categorical binary, n (%)				
None	1001/2351 (43%)	359/871 (41%)	1360/3222 (42%)	P/I: 1.1 (0.9, 1.3)
≤ 3 days per week	1042/2351 (44%)	370/871 (43%)	1412/3222 (44%)	P/I: 1.1 (0.9, 1.4)
> 3 days per week	308/2351 (13%)	142/871 (16%)	450/3222 (14%)	P/I: 0.8 (0.6, 1.1)

Significant differences in bold. Missing values excluded from denominator when calculating percentages. Some percentages may not total 100% due to rounding. Median (IQR) reported for count variables only. IQR = Interquartile range

^a^Estimates are unstandardised effects size for numeric variables and odds ratios for categorical or ordinal variables: ‘P-I’ is estimated difference Prescribed group—Illicit group, ‘P/I’ is odds ratio Prescribed/Illicit

^b^Includes non-binary, ‘prefer not to say’ and other identified genders

^c^In relationship includes defacto and married; Not currently in a relationship includes separated, divorced, widowed

^d^Includes both trade/vocational and undergraduate/postgraduate university qualifications

^e^Employed include full-time and part-time work; Not employed Includes home duties, students, unemployed, retired, and on disability pension

^f^Social media = Facebook, Twitter, Instagram, Snapchat; Online forum = Reddit, Whirlpool, Bluelight; Other sources = friend, consumer support group, Lambert Initiative website, traditional media (radio, tv, newspaper)

### Health conditions

The main health condition that respondents most commonly treated with medical cannabis was pain (37% [1171/3193]), followed by mental health/substance use (36% [1138/3193]) and sleep (15% [490/3193]). Prescribed users were significantly more likely to report that the main condition they treated with medical cannabis was a pain (OR = 1.3; CI 1.1, 1.5) or sleep (OR = 1.4; CI 1.1, 1.7) condition and significantly less likely to report their main condition was mental health/substance use (OR = 0.8; CI 0.7, 0.9; see Table 2). Prescribed users were also less likely to report their main condition was cancer although there were not many respondents with this condition (*n* = 35) and hence less certainty around this estimate (OR = 0.4; CI 0.2, 0.9).

**Table 2:** Main conditions treated with prescribed vs illicit medical cannabis

	Prescribed (*n* = 2331)	Illicit (*n* = 862)	Total (*N* = 3193)	OR (95% CI)
Pain	888 (38%)	283 (33%)	1171 (37%)	**P/I: 1.3 (1.1, 1.5)**
Mental health/substance Use	795 (34%)	343 (40%)	1138 (36%)	**P/I: 0.8 (0.7, 0.9)**
Sleep	381 (16%)	109 (13%)	490 (15%)	**P/I: 1.4 (1.1, 1.7)**
Neurological	105 (5%)	53 (6%)	158 (5%)	P/I: 0.7 (0.5, 1.1)
Gastrointestinal	52 (2%)	18 (2%)	70 (2%)	P/I: 1.1 (0.6, 2.0)
Cancer	19 (1%)	16 (2%)	35 (1%)	**P/I: 0.4 (0.2, 0.9)**
Other	91 (4%)	40 (5%)	131 (4%)	P/I: 0.8 (0.6, 1.3)

Significant differences in bold

Over 90% of respondents (2908/3193; illicit: 755/862 [88%]; prescribed: 2153/2331 [92%]) had received a diagnosis for the main condition they treated with medical cannabis, however the odds of having received a diagnosis were an estimated 70% higher for prescribed than illicit users (OR = 1.7, CI 1.3, 2.2).

There was no between-group difference in respondents’ ratings of the effectiveness of their medical cannabis, with < 1% (24/3193) reporting their condition was worse, 3% (85/3193) reporting no change and 97% (3084/3193) reporting improvement (824/862 [96%] illicit; 2260/2331 [97%] prescribed). There was also no significant difference in odds of reporting a greater amount of improvement between respondents who said their main condition was pain (96% [1122/1171] reported improvement), mental health (98% [1110/1138] reported improvement), or sleep (98% [480/490] reported improvement).

### Cannabis use

Prescribed medical cannabis users started using cannabis regularly for medical reasons significantly later in life (estimate = 4.3 years; CI 3.2, 5.5) than illicit users, although there was no significant between-group difference in age of onset of regular non-medical cannabis use (estimate = −0.3 years; CI − 1.2, 0.6; see Table 3). The prescribed group also used cannabis on significantly more days for medical reasons in the previous 28 days than the illicit group (estimate = 2.9 days; CI 2.2, 3.3) but fewer days for non-medical reasons (estimate = − 4.9 days; CI − 5.6, -4.2). Prescribed users were less likely to have used cannabis for non-medical reasons before they started using it medically (OR = 0.5; CI 0.5, 0.7), and more likely to use cannabis solely for medical reasons (OR = 3.0; CI 2.4, 3.6). Prescribed users paid $15.20 more on average each week for their medical cannabis (CI $7.4, $23.0) than illicit users; however, if respondents who did not pay for their medical cannabis—which was far more common in the illicit (24% [659/871]) than the prescribed (1% [31/2352]) group—were excluded from the analysis, prescribed users paid $10.40 dollars less than illicit users (CI − $19.1, − $1.7) (Table 3).

**Table 3:** Current and lifetime patterns of cannabis use

Characteristic	Prescribed	Illicit	Total	Comparisons estimate (95% CI)^a^
Never used cannabis use before medical use, categorical binary, n (%)	624/2352 (27%)	145/871 (17%)	769/3223 (24%)	**P/I: 0.6 (0.5, 0.7)**
Age first regular cannabis use for medical reason, numeric, (*N* = 3082)				
Mean (SD)	38.3 (13.9)	34.0 (15.2)	37.6 (14.4)	**P–I: 4.3 (3.2, 5.5)**
Median (IQR)	37 (28, 48)	30 (21, 45)	35 (25, 47)	
Age first regular cannabis use for non-medical reason, numeric, (*N* = 2452^b^)				
Mean (SD)	18.0 (10.8)	18.3 (10.8)	18.1 (10.8)	P–I: − 0.3 (− 1.2, 0.6)
Median (IQR)	18 (15, 22)	18 (15, 22)	18 (15, 22)	
Number of days in previous 28 used cannabis for medical reasons, numeric, (*N* = 3223)				
Mean (SD)	23.5 (7.9)	20.6 (9.9)	22.7 (8.6)	**P–I: 2.9 (2.2, 3.5)**
Median (IQR)	28 (20, 28)	25 (14, 28)	28 (20, 28)	
Number of days in previous 28 used cannabis for non-medical reasons, numeric, (*N* = 2452^b^)				
Mean (SD)	2.9 (7.0)	7.9 (11.0)	4.4 (8.6)	**P–I: − 4.9 (− 5.6, − 4.2)**
Median (IQR)	0 (0, 2)	0 (0, 14)	0 (0, 4)	
Estimated proportion of cannabis use for medical reasons in last 12 months, numeric, Mean (SD), (*N* = 2454^b^)	90.0 (17.7)	78.0 (22.7)	85.7 (20.0)	**P–I: 11.0 (9.3, 12.7)**
100% of cannabis use for medical reasons, binary categorical, n (%), *N* = 2454^b^	849/1728 (49.1%)	179/726 (25%)	1028/2454 (42%)	**P/I: 3.0 (2.4, 3.6)**
Weekly cost of medical cannabis, numeric, in $, (*N* = 3223)				
Mean (SD)	$99.0 ($96.6)	$83.8 ($108.8)	$94.9 ($100.3)	**P–I: $15.2 ($7.4, $23.0)**
Weekly cost of medical cannabis with respondents who did not pay excluded, numeric, in $, (*N* = 2980)				
Mean (SD)	$102.3 ($96.6)	$110.7 ($112.5)	$102.6 ($100.4)	**P–I: − $10.4 (− $19.1, -$1.7)**

Significant differences in bold. Missing values excluded from denominator when calculating percentages. Some percentages may not total 100% due to rounding

^a^Estimates are unstandardised effects size for numeric variables and odds ratios for categorical or ordinal variables

^b^Only respondents who answered yes to the question ‘Have you ever used cannabis for non-medical reasons?’ could see and answer this question

Only 1% (25/2334) of prescribed users said they were unsure of the composition of their medical cannabis, compared with 23% (197/863) of illicit users (OR = 25.0; CI 16.7, 50.0). Six percent (136/2334) of prescribed users said the composition of their medical cannabis varied, compared with 28% (245/863) of illicit users (OR = 6.3; CI 5.0, 8.3). Among prescribed users—who use regulated, commercially available products with labelled compositions on the packaging and can accurately ascertain the content of their medication—17% (380/2257) said they used THC-only products, 44% (993/2257) used products with mainly THC and a small amount of CBD, 25% (560/2257) a 1:1 THC/CBD formulation, 8% (183/2257) mostly CBD and a small amount of THC, and 6% (141/2257) CBD only. For those respondents who believed they knew the composition of their main source of medical cannabis (i.e. who did not select “I don’t know” in response to this question), the odds of prescribed users having a higher proportion of CBD in their main source of medical cannabis were 42% greater than illicit users (OR = 1.4; CI 1.2, 1.7).

**Fig. 1:**
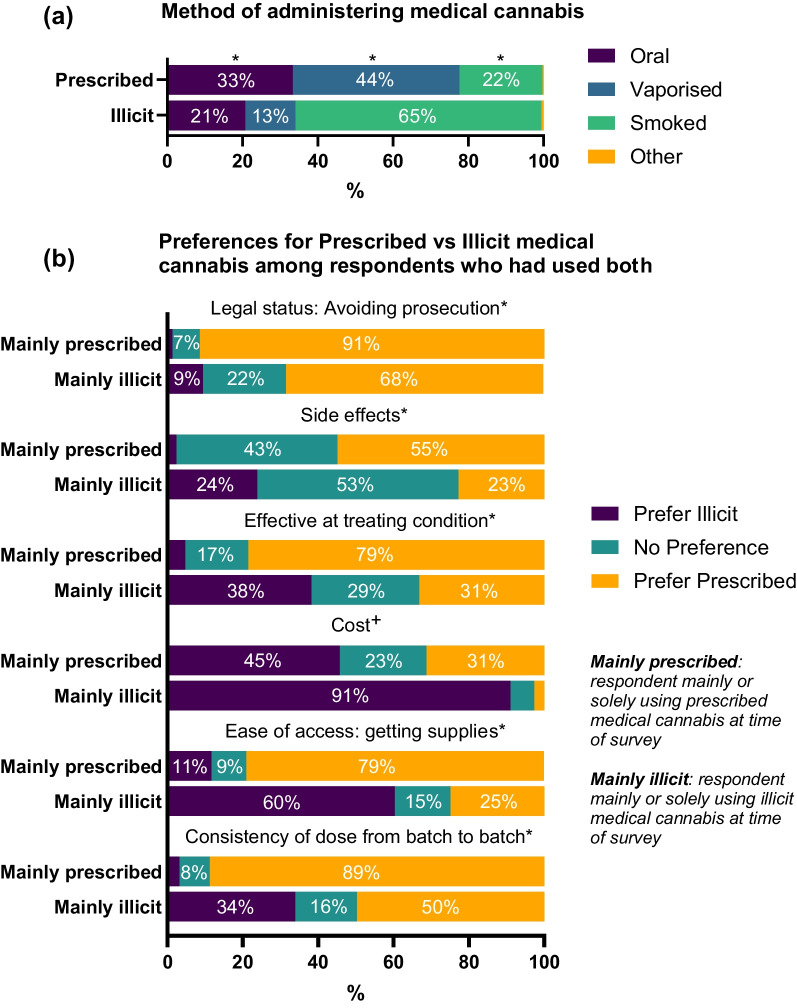
**a** Method of consumption in Prescribed and Illicit users. Asterisks above a colour indicate significant difference in proportion between Prescribed versus Illicit users for the method in question (indicated by matching colour). **b** Preferences for incongruent form of medical cannabis among respondents who had used both. * = Illicit significantly more likely to indicate incongruent preference, + = Prescribed more likely

A significantly greater proportion of prescribed users administered their medical cannabis mainly via oral (33% prescribed vs 21% illicit; OR = 1.9; CI 1.5, 2.4; Fig. 1a) or vaporised (44% prescribed vs 13% illicit; OR = 5.2; CI 4.0, 6.8) routes compared to illicit users, and a significantly lower proportion via smoking (22% prescribed vs 65% illicit; OR = 0.2; CI 0.1, 0.2).

### Side effects

The most common side effects reported by respondents were “dry mouth” (1917/3044 [63%]), “increased appetite” (1731/3044 [57%]), “drowsiness” (1529/3044 [50%]), “eye irritation” (965/3044 [32%]), and “memory problems” (914/3044 [30%]), with the overwhelming majority rating these as ‘mild and tolerable’ and never more than 3% of the cohort rating any symptom as ‘severe and intolerable’. Prescribed users reported significantly less severe “decreased appetite”, “dehydration”, “depression”, “fatigue”, “gastrointestinal complaints”, “headaches”, “nausea”, “panic”, “residual bad taste in mouth”, “respiratory complaints”, “sleep disturbance”, “sweating”, and “tremors” and significantly more severe “drowsiness” than illicit users (see Additional file 1: eTable S1). The median number of side effects of any severity was 4 (IQR: 2, 7; prescribed median = 4 [IQR: 2,7]; illicit median = 4 [IQR: 2, 8]). We calculated how many side-effects (either mild and tolerable or severe and intolerable) each respondent reported and estimated group differences in these counts. Prescribed users reported significantly fewer side effects, with the odds of prescribed users reporting any given side-effect an estimated 7.1 times lower than illicit users (OR = 0.1; CI 0.1, 0.2).

### Engaging with the healthcare system

Around 80% of survey respondents (1860/2334) who had been prescribed medical cannabis received their prescription from a GP or a specialist at a ‘cannabis clinic’ (focusing on medical cannabis treatment), with 20% (474/2334) obtaining their prescriptions from doctors in a general health setting. Respondents who were currently prescribed medical cannabis reported having been prescribed cannabis for a relatively short duration of 1.34 ± 1.2 years (Median [IQR] = 1 [0.5, 2]).

### Preferences for prescribed versus illicit medical cannabis

There were 1589 respondents who indicated that they had used both prescribed and illicit cannabis in the past. Of these ‘dual-users’, 88% (1400/1589) indicated that over the previous 12 months they had solely or mainly used prescribed medical cannabis while 12% (189/1589) had solely or mainly used illicit medical cannabis. This large difference in numbers reflects the fact that 78% [682] of the 871 respondents in the illicit group indicated they had *never* used prescribed products, compared with only 40% [952/2352] of the prescribed group indicating they had never used illicit products.

We asked these dual users whether they preferred prescribed or illicit cannabis across six domains, presented in Fig. 1b. We created a dichotomous variable for each of the six domains, indicating whether the dual-user stated a preference (either prefer or strongly prefer) that was incongruent with the type of cannabis (prescribed vs illicit) they were mainly using at the time. For example, if a dual-user who was mainly using prescribed cannabis indicated that they preferred illicit medical cannabis for ease of access this would constitute an incongruent preference. Dual-users who were currently using mainly prescribed medical cannabis had significantly lower odds of indicating a preference for illicit medical cannabis consistency (OR = 0.03; CI 0.02, 0.05), ease of access (OR = 0.40; CI 0.28, 0.58), effectiveness (OR = 0.10; CI 0.07, 0.14), side effects (OR = 0.08; CI 0.05, 0.13), and illegality (OR = 0.01; CI 0.00, 0.01) than the odds of illicit dual-users indicating a preference for prescribed. In contrast, these same dual-users using mainly prescribed medical cannabis had significantly greater odds of preferring illicit medical cannabis for its cost than dual-users currently using mainly illicit medical cannabis had of preferring prescribed (OR = 25.6; CI 11.3, 58.1).

## Discussion

The results of the CAMS-22 survey further underline the dramatic recent increase in the proportion of people using legally-prescribed rather than illicitly-sourced medical cannabis, consistent with recent TGA data indicating dramatic growth in the number of medical cannabis prescriptions in Australia since 2019 [8, 9, 16]. Almost three quarters of our sample had accessed mainly prescribed cannabis—compared with just over a third in our previous CAMS-20 survey [30], and less than 3% in our 2016 and 2018 surveys [4, 29]—allowing us for the first time to report the experiences of a large number of people who had been prescribed medical cannabis.

In CAMS-22, prescribed medical cannabis users appeared to have a different ‘profile’ than illicit users: they were more likely to be male, employed, and have tertiary qualifications. In addition, prescribed users tended to have started using cannabis for medical reasons later in life, used less cannabis for non-medical reasons, and were less likely to have used cannabis non-medically before commencing medical use. These differences suggest that for some illicit users the demarcation between non-medical and medical use is less clear than for prescribed users.

Medical cannabis—both illicit and prescribed—was primarily being used by respondents to treat pain (37%), mental health (36%) and sleep conditions (15%), consistent both with previous Australian surveys [4, 29] and with findings from studies of medical cannabis users around the world [18–20]. However, there were differences between prescribed and illicit users concerning which conditions they were more likely to be treating, with prescribed users more likely to use cannabis to treat a pain condition and less likely to treat a mental health condition than illicit users [29]. This is encouraging as there is better evidence for the effectiveness of cannabis for treating pain than for treating mental health conditions [1, 2, 38, 39]. Nonetheless, mental health conditions remain the second most commonly identified group of conditions treated with medicinal cannabis. The differences in main condition treated between prescribed and illicit users may stem from prescribed users’ receiving guidance from trained health professionals whose job is to be informed about the existing evidence for clinical indications for medications. Results from a recent survey of *n* = 505 Australian general practitioners support this interpretation, with respondents indicating they are much less comfortable with the idea of prescribing cannabis for anxiety, depression and sleep conditions than they are for pain, epilepsy, and chemotherapy-induced nausea [21]. Alternatively, it may be that the type of person who is already well-informed about the clinical evidence for effectiveness of cannabis for certain conditions is also more likely to seek their medical cannabis through legal channels. Unfortunately our survey is unable to give definitive answers on the reasons for the differences in conditions treated; future research among both clinicians and consumers may be able to explore these reasons in greater depth.

In CAMS-22 prescribed users were significantly more likely than illicit users to be mainly treating a sleep condition, reflecting the large increase in the proportion of SAS-B approvals granted for sleep disturbances in the last two years [16]. As with mental health disorders, there is scarce empirical evidence that medical cannabis is efficacious for sleep disorders [40–42], once again raising concerns over the apparent willingness of many Australian clinicians to prescribe medical cannabis for disorders for which there is limited evidence of efficacy.

Both prescribed and illicit users overwhelmingly endorsed the effectiveness of their medical cannabis in treating their main health condition. Over 95% of respondents who reported their main indication was pain, mental health, or sleep indicated that their condition was a little, much, or very much better since starting medical cannabis. In contrast to these overwhelmingly positive consumer reports, empirical evidence concerning efficacy of cannabis for mental health or sleep conditions is weak, and even for pain conditions, the available evidence suggests medicinal cannabis has modest efficacy [1, 2, 38, 43]. Recent observational and longitudinal studies of patients attest to a more general improvement in health-related quality of life in patients prescribed medical cannabis, seemingly irrespective of whether their primary condition is being specifically treated [44–47]. It may be that cannabis leads to a shift in hedonics, and broader health and social wellbeing, rather than specific clinical outcomes such as pain intensity or sleep duration.

The disparity between self-reported effectiveness of medical cannabis from patient surveys and cohort studies compared to evidence from ‘gold standard’ randomised trials underscores many of the challenges facing consumers, clinicians, regulators and researchers. For many clinical conditions the efficacy of medical cannabis has not been studied thoroughly, with the majority of studies being cross-sectional surveys or longitudinal cohort studies [1, 2, 38–42]. Such studies: (1) lack comparison groups and therefore provide lower quality evidence than randomised trials, and (2) are subject to limitations in interpretation including the potential for patient and/or clinician expectancy/placebo effects and often a ‘survivor bias’ caused by failure to include data from patients who discontinued treatment due to limited effectiveness or unpleasant side effects [48, 49]. For mental health or sleep conditions, the second and third most common conditions respondents treated with medical cannabis, randomised controlled trials are either underpowered, suffer from methodological limitations that hamper meta-analyses, or simply have never been conducted [38–42]. In addition, studies typically exclude patients with multiple comorbidities limiting generalisability of experimental findings to real world patients. There is a need for consensus approaches to undertaking clinical trials with medicinal cannabis products to better allow pooling of data across trials. In addition to their clinical endpoints, future clinical trials should incorporate patient-reported outcome measures that capture ‘what is important to patients’, so as to disentangle the seeming contradiction between what patients report and researchers find.

Whilst we advocate for more and better-conducted research, patients, clinicians, and regulators should address this evidence gap in the ‘here and now’. There is greater need for independent consumer education that is not led by the medical cannabis industry, with more explicit information about safety and effectiveness of using different medical cannabis products for different conditions. Similarly, in most settings, medical cannabis is prescribed ‘off label’. Healthcare professionals need to consider how they undertake ‘off label’ prescribing of unregistered medicines while abiding by local professional standards [50]. There are important issues surrounding: how prescribers of medical cannabis obtain and document informed consent; the role of published clinical guidance; seeking second opinions with relevant specialists; and understanding medico-legal issues such as insurance. Regulators must also consider the level of autonomy they wish to allow clinicians. For example, in some jurisdictions regulators limit the range of cannabis products or conditions that a doctor can prescribe for, while in others, such as Australia, the regulatory framework leaves this to the discretion of the clinician and patient [3].

Leaving aside the issue of efficacy, our findings suggest that people who are using prescribed medical cannabis may experience health benefits over people who self-medicate with illicit cannabis. Prescribed users reported experiencing significantly fewer side-effects than illicit users, were more likely to consume their medical cannabis via safer routes (oral or vaporised rather than smoking) and consumed significantly less tobacco. Prescribed users were also significantly more certain of the composition of their medical cannabis, exposing them to less risk of harm via under- or overdosing or unknowingly ingesting harmful contaminants.

This iteration of the CAMS survey was the first with a sufficient number of respondents who had used both prescribed and illicit cannabis to allow for an analysis of their preferences. Among these ‘dual-users’, respondents were far more likely to prefer prescribed medical cannabis for its consistency of dose, ease of getting supplies, effectiveness in treating their condition, better side-effects profile, and reduced risk of legal issues. This highlights the benefits to patients of being able to access medical cannabis of known potency through legal channels rather than having to obtain it from illicit sources. The only feature in which dual-users preferred illicit cannabis was with respect to cost, and many users of illicit medical cannabis have cited this as a barrier to seeking a prescription [4, 29, 30]. Interestingly however, these user preferences may not reflect reality, for when we excluded respondents who did not pay for their illicit medical cannabis (e.g. who grew their own or were given cannabis as gifts) prescribed users actually paid *less* on average per week than illicit users. This departure from earlier CAMS surveys may reflecting the diminishing cost of THC-based medical cannabis products as supply has increased and access has improved, though no studies have yet published reliable figures of how costs have changed over time.

There were several limitations to the CAMS-22 survey that may have biased results. First, self-report inevitably leads to inaccuracies in data, whether due to mistaken recall, lack of insight into effectiveness, fatigue in later sections of the survey, or incorrect reporting of diagnostic conditions and products used. Second, any research method that uses convenience sampling, such as an anonymous online survey, is likely to suffer from selection bias, with people who have had positive experiences with medical cannabis, or who have personal or political agenda promoting medical cannabis, more inclined to respond than those who have had negative experiences or who disapprove of medical cannabis. Such bias can significantly limit the generalisability of any findings [51], hence to what extent our results are representative of the general population of Australian medical cannabis users remains unclear. Third, naturally a higher proportion of the 14% of respondents who were recruited through medical cannabis providers were prescribed users (18% vs 4% illicit users). This imbalance may have biased sampling and affected the generalisability of our results. Finally, the main sources of recruitment have changed with each iteration of CAMS (e.g. CAMS-20 recruited mainly through Twitter whereas CAMS-22 recruited mainly through Facebook), meaning that changes across surveys may reflect changes in the sample rather than trends over time in the broader community.

## Conclusion

From a harm reduction perspective, there is much to recommend prescribed over illicit medical cannabis: it tends to be administered via safer routes, appears to have less severe side-effects, reduces the risk of entanglement with the legal system, gives consumers more certainty regarding contents and dose, and increases the chances that people will engage with health professionals around how best to treat their health condition. However, further high-quality clinical trials and stronger research evidence is required to establish the role of different medical cannabis preparations in treating the wide array of conditions for which medical cannabis is being used—particularly of the many medical conditions for which supporting evidence remains sparse.

### Supplementary Information

**Additional file 1.** Rates of Side effects and full CAMS-22 questionnaire.

## Data Availability

The survey questions are available as an online supplement. For proprietary reasons datasets will not be made publicly available, however the data and code used for these analyses will be made available from the corresponding author on request.
